# Generation, Characterization, and Application of Inducible Proliferative Adult Human Epicardium-Derived Cells

**DOI:** 10.3390/cells10082064

**Published:** 2021-08-12

**Authors:** Yang Ge, Anke M. Smits, Jia Liu, Juan Zhang, Thomas J. van Brakel, Marie José T. H. Goumans, Monique R. M. Jongbloed, Antoine A. F. de Vries

**Affiliations:** 1 Department of Anatomy & Embryology, Leiden University Medical Center, Einthovenweg 20, 2333 ZC Leiden, The Netherlands; y.ge@lumc.nl (Y.G.); m.r.m.jongbloed@lumc.nl (M.R.M.J.); 2 Department of Cardiology, Leiden University Medical Center, Albinusdreef 2, 2333 ZA Leiden, The Netherlands; liujia0702@gmail.com (J.L.); J.Zhang@lumc.nl (J.Z.); A.A.F.de_Vries@lumc.nl (A.A.F.d.V.); 3 Department of Cell and Chemical Biology, Leiden University Medical Center, Einthovenweg 20, 2333 ZC Leiden, The Netherlands; a.m.smits@lumc.nl; 4 Central Laboratory, Longgang District People’s Hospital of Shenzhen & The Third Affiliated Hospital of The Chinese University of Hong Kong, Shenzhen 518172, China; 5 Department of Cardiothoracic Surgery, Leiden University Medical Center, Albinusdreef 2, 2333 ZC Leiden, The Netherlands; thomas.v.brakel@catharinaziekenhuis.nl

**Keywords:** epicardium-derived cells (EPDCs), conditional immortalization, simian virus 40 large T antigen (LT), epithelial-to-mesenchymal transition (EMT)

## Abstract

Rationale: In recent decades, the great potential of human epicardium-derived cells (EPDCs) as an endogenous cell source for cardiac regeneration has been recognized. The limited availability and low proliferation capacity of primary human EPDCs and phenotypic differences between EPDCs obtained from different individuals hampers their reproducible use for experimental studies. Aim: To generate and characterize inducible proliferative adult human EPDCs for use in fundamental and applied research. Methods and results: Inducible proliferation of human EPDCs was achieved by doxycycline-controlled expression of simian virus 40 large T antigen (LT) with a repressor-based lentiviral Tet-On system. In the presence of doxycycline, these inducible EPDCs (iEPDCs) displayed high and long-term proliferation capacity. After doxycycline removal, LT expression ceased and the iEPDCs regained their cuboidal epithelial morphology. Similar to primary EPDCs, iEPDCs underwent an epithelial-to-mesenchymal transition (EMT) after stimulation with transforming growth factor β3. This was confirmed by reverse transcription-quantitative polymerase chain reaction analysis of epithelial and mesenchymal marker gene expression and (immuno) cytochemical staining. Collagen gel-based cell invasion assays demonstrated that mesenchymal iEPDCs, like primary EPDCs, possess increased invasion and migration capacities as compared to their epithelial counterparts. Mesenchymal iEPDCs co-cultured with sympathetic ganglia stimulated neurite outgrowth similarly to primary EPDCs. Conclusion: Using an inducible LT expression system, inducible proliferative adult human EPDCs were generated displaying high proliferative capacity in the presence of doxycycline. These iEPDCs maintain essential epicardial characteristics with respect to morphology, EMT ability, and paracrine signaling following doxycycline removal. This renders iEPDCs a highly useful new in vitro model for studying human epicardial properties.

## 1. Introduction

The cardiac outer layer, or epicardium, is composed of multifunctional and multipotent cells with important roles during fetal development [1,2,3]. In the healthy adult heart, epicardial cells are quiescent and have a squamous (flat) morphology. Studies in animal models have shown that epicardial cells become activated in response to pathological triggers, such as myocardial infarction [4,5,6]. Epicardial cells then adopt a cuboidal appearance, undergo epithelial-to-mesenchymal transition (EMT), migrate into the subepicardial space, and subsequently into the myocardium [4]. After migration, epicardial cells are referred to as epicardium-derived cells (EPDCs). These EPDCs can differentiate into coronary smooth muscle cells and cardiac fibroblasts [5,7]. In addition, EPDCs have strong paracrine effects, and factors secreted by EPDCs have been shown to promote angiogenesis, reduce infarct size, and improve cardiac function [8].

Given the ability of EPDCs to (i) contribute to different cardiac cell lineages, (ii) produce beneficial paracrine factors for heart development and (iii) recapitulate an embryonic epicardial phenotype after cardiac damage in adult hearts, they are a highly interesting study object from a cardiac developmental and regenerative perspective. An increased understanding of epicardial cell properties and behavior could not only provide new insights into certain (congenital) heart diseases but also generate additional knowledge about the ability of epicardial cells to participate in human cardiac repair [9,10]. Our research group has previously established cell culture models based on primary human EPDCs that allowed us to investigate processes like EMT [11,12,13]. Furthermore, we found that the injection of human EPDCs into mouse infarcted myocardium improves cardiac function, leads to scar reduction, and induces angiogenesis likely though a paracrine mechanism [5,14,15]. Co-culturing human EPDCs with neuronal tissue showed that human EPDC-derived factors can influence the outgrowth of cardiac autonomic ganglia [16]. Human epicardial cells thus hold strong potential for cardiac regeneration therapy, but there are several challenges and pitfalls when using these cells. First, the relative scarcity of human epicardium and EPDC sources hampers the deeper investigation of EPDC function and their potential for cardiac repair. Furthermore, although we developed a reliable method for the isolation and expansion of EPDCs from human specimens [17], EPDCs possess limited proliferation capacity and tend to lose their epithelial phenotype within a few passages. In addition, the application of EPDCs derived from different patients and with different passage numbers, has likely contributed to the variable results obtained in in vitro experiments. These considerations inspired us to investigate whether conditional immortalization would allow the generation of lines of highly proliferative adult human EPDCs with preserved functionality. At present, the only immortalized EPDCs available are of rodent origin, isolated from transgenic mouse embryos obtained by crossing wild type or Sm22α-lacZ mice with the ImmortoMouse line, which harbors a H-2K^b^ promoter-driven expression unit directing the synthesis of a temperature-sensitive version of the simian virus 40 (SV40) large T antigen (LT) [18,19,20]. This is the first study to generate a polyclonal line of human EPDCs generated by applying a lentiviral vector (LV) expressing wild type LT in a doxycycline (Dox)-dependent manner. We designated the resulting cells inducible EPDCs (iEPDCs) and studied their proliferation capacity, their LT expression in the presence and absence of Dox, and their expression of epicardial genes after treatment with modulators of transforming growth factor β (TGFβ) signaling. In addition, known functions of EPDCs, including EMT, migration and invasion, and simulation of neurite outgrowth, were explored to determine whether iEPDCs maintain the phenotypic and functional properties of the primary cells from which they are derived.

## 2. Methods

### 2.1. LV Production

Primary human EPDCs were conditionally immortalized using the self-inactivating, vesicular stomatitis virus G protein-pseudotyped LV named LV.iHsUBC.LT-WT (for a map of the corresponding LV shuttle plasmid, see Supplemental Figure S1). LV.iHsUBC.LT-WT directs the Dox-dependent expression of wild type SV40 LT and only differs from the previously described LV.iHsUBC.LT-*ts*A58 [21] by harboring the coding sequence of wild type SV40 LT instead of the temperature-sensitive SV40 LT mutant *ts*A58. The production, purification, and concentration of LV particles was done essentially as described in [22].

### 2.2. Isolation and Culture of Primary Human EPDCs

Human EPDCs were isolated from adult human atrial samples (auricles), that were strictly anonymously collected as surgical waste material. Tissues were delivered to the researcher without any additional information to guarantee the full anonymity of the tissues so as not to include or identify human subjects. This study was conducted in accordance with the Ethical Principles of the Declaration of Helsinki 2013 and according to the Dutch regulation for responsible use of human tissues for medical research purposes. The institutional Medical Ethics Committee ruled that the Medical Research Involving Human Subject Act (WMO) does not apply to the use of surgical waste material (reference number B12.017). Human EPDCs were dissociated from the specimens as previously described [17]. Briefly, the epicardium was carefully removed from the underlying myocardium and cut into small pieces followed by three rounds of 0.25% Trypsin-EDTA (25200056; Thermo Fisher Scientific, Bleiswijk, The Netherlands) incubation at 37 °C for 30 min in total. After digestion, the cell suspension was passed through a series of syringes of decreasing internal diameter (19G to 22G) and through a 100-μm BD Falcon cell strainer (BD Biosciences, Vianen, The Netherlands). Next, the cells were plated on dishes coated with 0.1% porcine gelatin (G1890; Sigma-Aldrich, St. Louis, MO, USA). Successful isolation of epithelial EPDCs was indicated by the recovery of cells with a cuboidal morphology. EPDCs were cultured in complete medium supplemented with the ALK5 kinase inhibitor SB431542 (SB, 10 μM; Tocris Bioscience, Ellisville, MO, USA) to maintain an epithelial state. Complete medium is a 1:1 mixture of low glucose Dulbecco’s modified Eagle’s medium (10567014; Thermo Fisher Scientific) and medium 199 (31150022; Thermo Fisher Scientific) with 10% heat-inactivated fetal bovine serum (FBS; S1860-500; Biowest, Nuaillé, France) and 1% 100× penicillin/streptomycin solution (15140122; Thermo Fisher Scientific).

### 2.3. Transduction of Human EPDCs

The primary human EPDCs were first expanded for three passages to create a seed stock. Next, the cells were transduced with LV.HsUBC.LT-WT in the presence of 5 µg/mL of DEAE-dextran (4198; Carl Roth, Karlsruhe, Germany) [23] to facilitate vector uptake. Twenty-four hours after transduction, the LV-containing medium was replaced by complete medium supplemented with 10 μM SB and 50 ng/mL Dox (D9891; Sigma-Aldrich) to induce LT expression. The medium containing SB and Dox was refreshed every two days. LT expression in iEPDCs in the presence of Dox was visualized by immunofluorescence staining.

### 2.4. Analysis of Cell Growth of Primary and Transduced EPDCs

Both primary and transduced EPDCs were cultured in medium supplemented with SB and 50 ng/mL Dox. When the cultures approached confluence, the cells were passaged at a splitting ratio of 1:8 (iEPDCs) or 1:2 (primary EPDCs). To compile cell growth curves, numbers of population doublings (PDs) were plotted against days in culture until the EPDCs lost their epithelial phenotype (i.e., cobblestone cell morphology).

**Figure 1:**
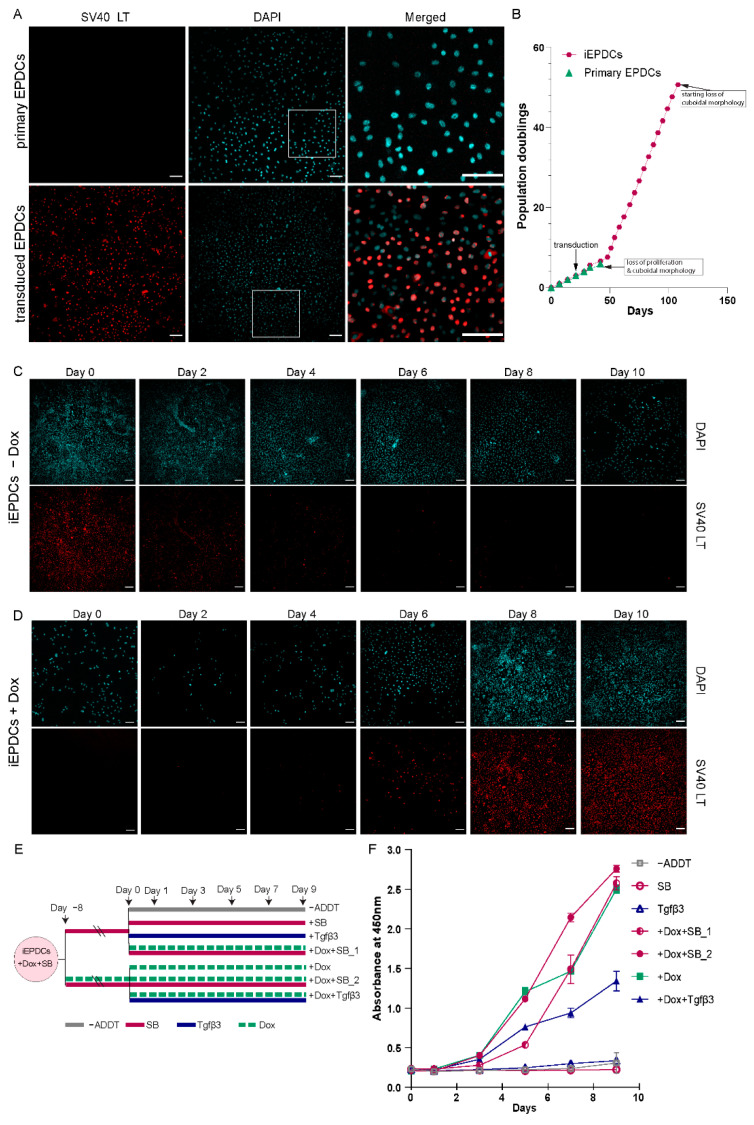
Generation of iEPDCs. (**A**) Confirmation of LT expression in iEPDCs (PD10) in the presence of Dox by immunofluorescent staining with anti-SV40 LT. (**B**) Growth curves of primary EPDCs and iEPDCs cultured in the presence of Dox. Recordings were ended when the EPDCs started to lose their cuboidal epithelial morphology. (**C**,**D**) Timeline of LT expression in iEPDCs after switching from Dox-containing to Dox-free culture medium (**C**) and vice versa (**D**). (**E**) Overview of the different pretreatments and culture conditions to which the iEPDCs were exposed for cell proliferation analysis. (**F**) Quantification of the proliferation of iEPDCs under the conditions indicated in (**E**). Scale bar = 100 µm. −ADDT = no additive.

To assess the cell proliferation rate of iEPDCs under different conditions, a Cell Counting Kit-8 (Dojindo Molecular Technologies, Rockville, MD, USA) was applied according to the manufacturer’s protocol using cells of PD28. The iEPDCs were first cultured in complete medium supplemented with Dox and SB or only with SB for eight days. Next, the cells were seeded into 96-well culture plates at a density of 1000 cells/well and exposed to different culture conditions. At culture day 0, 1, 3, 5, 7, and 9, the production of WST-8 formazan in each well (*n* = 3 per condition) was measured as an indicator of viable cell number (Figure 1E).

### 2.5. Inducible LT Expression upon Dox Addition

To study the dynamical changes in LT expression following Dox removal, iEPDCs that had been maintained in complete medium with SB and Dox were seeded into a 96-well culture plate and given complete medium supplemented with SB but without Dox. After the indicated times of culture in Dox-free medium, the cells were fixed with 4% paraformaldehyde (104005; Merck Millipore, Darmstadt, Germany) for subsequent immunostaining with an LT-specific monoclonal antibody (see below). In parallel, iEPDCs that had been kept in Dox-free medium for 8 days were seeded into a 96-well culture plate and exposed for different time periods to Dox-containing complete medium with SB before being fixed for anti-LT staining.

### 2.6. EMT Assay

Primary EPDCs and iEPDCs were seeded in 96-well plates (for staining) and 6-well plates (for RNA isolation). After five days of culture in Dox-free complete medium without additives or in Dox-free complete medium supplemented with either SB (10 μM; to maintain an epithelial phenotype) or TGFβ3 (1 ng/mL; to induce EMT), the cells were either processed for (immuno) cytochemical staining or used for RNA extraction. Total cellular RNA was isolated using the RNeasy Mini Kit (QIAGEN Benelux, Venlo, The Netherlands) according to the manufacturer’s instruction. Afterwards, cDNA was synthesized with the aid of the iScript cDNA Synthesis Kit (Bio-Rad Laboratories, Lunteren, The Netherlands). Reverse transcription-quantitative polymerase chain reactions (RT-qPCRs) were performed using iQ SYBR Green Supermix (Bio-Rad Laboratories) and the primer pairs (obtained from Integrated DNA Technologies, Leuven, Belgium) shown in Supplemental Table S1. The forward and reverse primer in each primer pair were targeting different exons and their specificity was confirmed by melting curve analysis and agarose gel electrophoresis of the amplification products. PCR amplification was performed in a *CFX384* Touch Real-Time PCR Detection System (Bio-Rad Laboratories). For each primer pair, the amplification efficiency was determined using serial dilutions of target RNAs. The three most stable reference genes were chosen based on geNORM [24]. For each sample and for each target gene, RT-qPCR was performed in triplicate and its expression was calculated using the amplification efficiency and normalized to the geometric mean of three reference genes (i.e., GAPDH, TBP, B2M) [24,25]. Depending on the experiment, samples were normalized to the SB-treated sample and results are shown as relative expression. Moreover, the RT-qPCR data of iEPDCs in Supplemental Figure S3 are average values derived from the analysis of cells at PD25, -28, -30, and -35, i.e., each bar in this figure represents four biological replicates.

### 2.7. Scratch Wound Healing Assay

Eight days after removal of Dox, the iEPDCs were cultured for five days in complete medium containing SB (10 μM) or TGFβ3 (1 ng/mL) to keep epithelial iEPDCs or obtain mesenchymal iEPDCs, respectively. Next, the iEPDCs were seeded into a 96-well culture plate for a scratch wound healing assay at a density of 20,000 cells/well. Twenty four hours before making a scratch, the epithelial iEPDCs were given complete medium with or without SB while the mesenchymal EPDCs received complete medium with or without TGFβ3. The IncuCyte S3 Live-Cell Analysis System (Sartorius Stedim Netherlands, Amersfoort, The Netherlands) was used to make the scratch and to image and analyze the cell migration according to the manufacturer’s instructions.

### 2.8. Invasion Assay

Eight days after removal of Dox, the iEPDCs were cultured for five days in complete medium containing SB (10 μM) or TGFβ3 (1 ng/mL) to keep epithelial iEPDCs or obtain mesenchymal iEPDCs, respectively. Next, aggregates of 20,000 epithelial iEDPCs, mesenchymal iEPDCs, or primary EPDCs with a total volume of 30 μL were formed by the hanging drop technique. Twenty-four hours before the invasion assay started, the epithelial iEPDCs in the hanging drop were given complete medium with or without SB while the clumps of mesenchymal EPDCs received complete medium with or without TGFβ3. To measure their invasion ability, aggregates of primary EPDCs and of iEPDCs were placed in drops of 3 mg/mL rat tail collagen I (354236; Corning Life Sciences, Amsterdam, The Netherlands) and cultured for the indicated time periods in EPDC culture medium. Time-lapse images were captured immediately with the EVOS FL Auto 2 Imaging System (Thermo Fisher Scientific). The invasion distances of the cells away from the aggregates were measured with ImageJ 1.52p (National Institutes of Health [NIH], Bethesda, MA, USA).

### 2.9. Co-Culture of Primary EPDCs and iEPDCs with Sympathetic Ganglia

To further test the experimental applicability of iEPDCs, co-cultures of mesenchymal iEPDCs with sympathetic ganglia (*n* = 10) were performed, as was done previously with primary EPDCs [16]. Superior cervical ganglia were isolated from (E) 18.5-day C57BL/6J mouse embryos (Charles River Laboratories, ‘s-Hertogenbosch, The Netherlands, *n* = 5) and co-cultured with mesenchymal EPDCs (primary EPDCs of PD8 or iEPDCs of PD30 that had been cultured without Dox for 15 days) as described previously [16]. Ganglionic outgrowth was determined after six days of co-culturing by immunochemical staining for β3 tubulin. All animal experiments were carried out according to the Guide for the Care and Use of Laboratory Animals published by the NIH and approved by the Animal Ethics Committee of the Leiden University Medical Center (Leiden, The Netherlands, license number AVD1160020185325).

### 2.10. Immunofluorescence Staining

Cells were fixed with 4% paraformaldehyde in phosphate-buffered saline (PBS), permeabilized with 0.5% Tween in PBS and non-specific epitopes were blocked with PBS containing 1% bovine serum albumin (A8022; Sigma-Aldrich) and 0.05% Tween 20 (822184, Merck Millipore, Darmstadt, Germany). The monocultures of EPDCs were stained with the following primary antibodies: mouse anti-SV40 LT (Santa Cruz Biotechnology, Heidelberg, Germany; SC-147; 1:400) or rabbit anti-Wilms tumor 1 (WT1, marker for epicardial tissues) (Abcam, Cambridge, MA, USA; ab89901; 1:100). The co-cultures of EPDCs and neurons were stained with rabbit anti β3 (Tubb3, marker for neural tissues) antibodies (Sigma-Aldrich; T2200; 1:500) overnight at 4 °C. After three rinses with 0.05% Tween 20 in PBS, bound primary antibodies were detected by incubation with Alexa Fluor 594-conjugated donkey anti-mouse IgG(H + L) (Thermo Fisher Scientific; A-21203; 1:250) or Alexa Fluor 488-conjugated donkey anti-rabbit IgG(H + L) (Thermo Fisher Scientific; A-21206; 1:250). For the detection of F-actin, Alexa Fluor 594-conjugated phalloidin (Thermo Fisher Scientific; A12381, 1:200) was used. Incubation with the secondary antibodies was done at room temperature and lasted for 1 h (monocultures) or for 2.5 h (co-cultures) and was followed by a single wash with PBS. DAPI (300 nM; D3571; Thermo Fisher Scientific) was used to stain nuclei. All images were captured with a Leica TCS SP8 confocal laser scanning microscope (Leica Microsystems, Wetzlar, Germany).

### 2.11. Statistics

Graphs are presented as mean ± standard error of the mean (SEM). Gene expression, cell migration, and invasion were compared using one-way analysis of variance (ANOVA) (plus Tukey’s multiple comparison test). The presence of neurite outgrowth in vehicle and EPDC co-culture groups was compared using a Chi-square test. GraphPad Prism (GraphPad Software, San Diego, CA, USA; version 8) was used for statistical analysis.

## 3. Results

### 3.1. Generation of iEPDCs

Primary adult human EPDCs at PD3 were transduced with an LV mediating Dox-dependent expression of wild type LT to generate a polyclonal line of EPDCs with inducible proliferation capacity (Supplemental Figure S1). Immunofluorescence staining of primary and transduced EPDCs that were cultured in the presence of Dox showed LT expression in the transduced EPDCs, while no LT was detected in the primary EPDCs (Figure 1A). In an attempt to preserve their epithelial phenotype, both the primary EPDCs and the iEPDCs were cultured in medium supplemented with the ALK5 kinase inhibitor SB to block endogenous TGFβ signaling. This, however, could not prevent the primary EPDCs from undergoing EMT after only a few cell divisions, causing them to lose their epithelial cobblestone-like morphology as early as at PD5 (Supplemental Figure S2). The phenotypic change of the primary EPDCs was accompanied by a strong reduction in proliferation capacity (Figure 1B). In contrast, the transduced cells (iEPDCs) were able to proliferate long-term without losing their cuboidal morphology. For iEPDCs, the earliest signs of EMT and loss of epithelial cobblestone-like morphology only occurred after PD51 (Figure 1B, Supplemental Figure S2A,B). These changes in iEPDC behavior coincided with an increase in the percentage of senescent cells as assessed by β-galactosidase staining (Supplemental Figure S2C).

To study the kinetics of Dox-dependent LT expression, iEPDCs were transferred from Dox-containing culture medium to Dox-free culture medium or vice versa and analyzed by immunostaining before (day 0) and 2, 4, 6, 8, and 10 days after medium change. While LT was highly expressed in iEPDCs cultured in the presence of Dox (Figure 1C, day 0), the omission of Dox resulted in a gradual decrease in the LT level in the first 4 days, and no obvious LT expression could be detected from day 6 onwards (Figure 1C). The addition of Dox to the culture medium of iEPDCs that had been maintained for 8 days in Dox-free culture medium induced LT expression. As is shown in Figure 1D, LT expression was first detectable 4 days after Dox addition and further increased afterwards to reach a plateau between day 8 and day 10 of culture in Dox-free medium.

Subsequently, the proliferation rate of iEPDCs in the presence and absence of Dox was quantified colorimetrically by measuring the bioreduction of WST-8. In primary adult human EPDCs, the addition of SB allows short-term in vitro expansion while maintaining their cuboidal epithelial morphology. Conversely, activation of the TGFβ signaling cascade in primary adult human EPDCs stimulates the transition from an epithelial to a mesenchymal cell type and inhibits epicardial cell proliferation. [26] We therefore also tested the effects of SB and TGFβ3 treatment on the proliferation capacity of iEPDCs cultured in the presence and absence of Dox, as shown in Figure 1E. Exposure of iEPDCs to Dox invariably resulted in cell proliferation with the highest proliferation rate observed in the cells that were also treated with SB. The addition of TGFβ3 to the culture medium stimulated iEPDCs to undergo EMT and lowered the cell proliferation induced by Dox (Figure 1F). In contrast to the iEPDCs that were cultured with Dox, iEPDCs did not show apparent proliferation when kept in Dox-free medium, even when not in the presence of SB (Figure 1F). Consistent with the kinetics of Dox-induced LT expression (Figure 1D), iEPDC proliferation started around four days after exposure to Dox (Figure 1F).

### 3.2. IEPDCs Undergo EMT and Show a Mesenchymal Phenotype upon TGFβ3 Stimulation

EMT of EPDCs starts with the loss of their cuboidal epithelial (cobblestone-like) phenotype and of their cell–cell junctions [4,27,28,29]. Subsequently, epithelial EPDCs acquire a spindle-shaped mesenchymal cell morphology and mesenchymal characteristics with upregulated expression of α-smooth muscle actin (ACTA2), fibronectin (FN1), collagen type I α1 (COL1A1) and N-cadherin (CDH2). The upregulation of mesenchymal markers is accompanied by downregulation of WT1, E-cadherin (CDH1), and other epicardial genes, like basonuclin1 (BNC1) [12,30,31] and aldehyde dehydrogenase 1 family member A2 (ALDH1A2) [32,33], which were previously found to be enriched in both developing epicardium and in vitro cultured primary adult human EPDCs.

**Figure 2:**
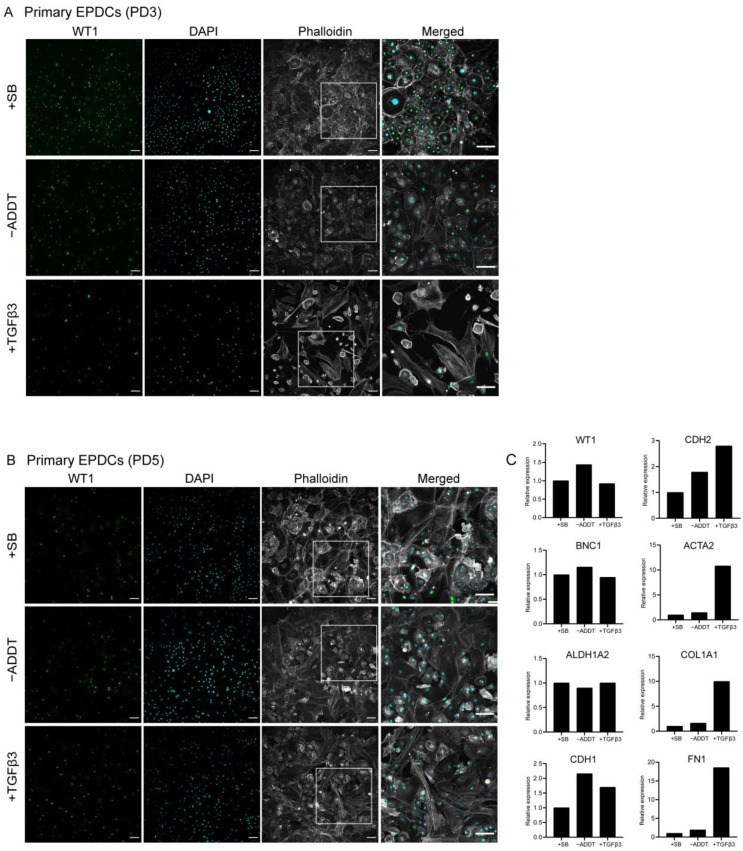
Primary human EPDCs undergo EMT and show a mesenchymal phenotype upon TGFβ3 stimulation. (**A**) Immunostaining showing that SB-treated primary human EPDCs of PD3 display a typical cuboidal epithelial morphology (indicated by phalloidin staining) and nuclear WT1 expression. TGFβ3-treated primary EPDCs undergo EMT, acquire a spindle-like appearance, which is accompanied by a large decrease in the percentage of WT1-positive cells. Culture without SB or TGFβ3 causes a small fraction of PD3 EPDCs to lose their cuboidal morphology and nuclear WT1 expression. (**B**) Immunofluorescent staining of primary human EPDCs of PD5 subjected to the same treatments as in (**A**) Irrespective of the specific culture conditions, primary human EPDCs of PD5 show a low percentage of WT1-positive cells and display signs of EMT. (**C**) Assessment by RT-qPCR of epithelial and mesenchymal marker gene expression in primary human EPDCs of PD5 cultured under different conditions. Technical replicates: 3, biological replicates: 1. Scale bar = 100 µm. −ADDT = no additive.

**Figure 3:**
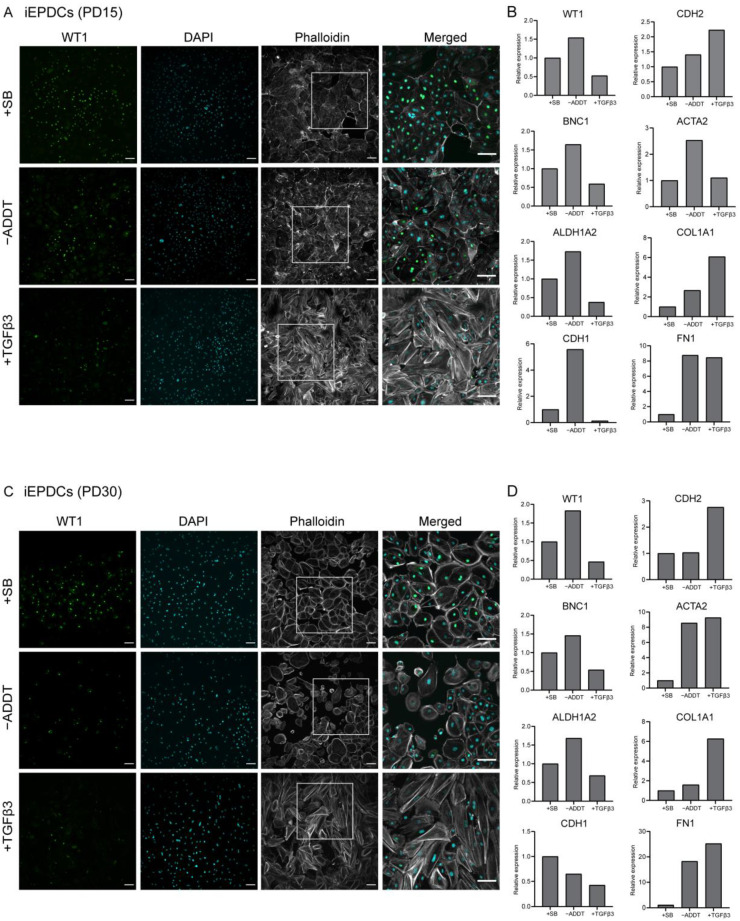
iEPDCs undergo EMT and show a mesenchymal phenotype upon TGFβ3 stimulation. (**A**). Immunostaining of iEPDCs of PD15 cultured in Dox-free medium containing SB (10 µM), TGFβ3 (1 ng/mL), or neither of the two. The iEPDCs show similar morphological characteristics and WT1 expression profiles as equally treated primary human EPDCs of PD3. (**B**) Assessment by RT-qPCR of epithelial and mesenchymal marker gene expression in iEPDCs of PD15 following different treatments. (**C**) Immunostaining of iEPDCs of PD30 cultured in Dox-free medium containing SB, TGFβ3, or neither of the two. The iEPDCs show similar morphological characteristics and WT1 expression profiles as equally treated primary human EPDCs of PD3. (**D**). Assessment by RT-qPCR of epithelial and mesenchymal marker gene expression in iEPDCs of PD30 following different treatments. Technical replicates: 3, biological replicates: 1. Scale bar = 100 µm. −ADDT = no additive.

To study the ability of iEPDCs to undergo EMT, we exposed iEPDCs of PD15 and -30 to Dox-free medium without additives (control conditions) or supplemented with either SB or TGFβ3 for five days and compared the cell morphology and the protein level of the epithelial marker WT1 with those of similarly treated primary EPDCs of PD3 and -5. To eliminate the possible effects of residual LT expression, Dox was removed from the iEPDC culture medium 4–8 days before SB or TGFβ3 supplementation. As expected, the primary EPDCs and iEPDCs displayed a cuboidal cell morphology and higher WT1 protein expression in the presence of SB than under control conditions (Figure 2A,B and Figure 3A,C). After culturing for five days in the absence of SB, neither primary EPDCs nor iEPDCs showed morphological signs of EMT (Figure 2A,B and Figure 3A,C). Upon TGFβ3 stimulation, both primary EPDCs and iEPDCs transformed into spindle-shaped mesenchymal cells and showed redistribution of F-actin as assessed by phalloidin staining from the cortical cytoskeleton to stress fibers, which is indicative of the occurrence of EMT (Figure 2A,B and Figure 3A,C). EMT was further confirmed in both primary EPDCs and iEPDCs by RT-qPCR analysis, which showed downregulation of epithelial marker genes like WT1 and BNC1 and upregulation of mesenchymal marker genes, e.g., ACTA2 and FN1, when comparing SB- with TGFβ3-treated cells (Figure 2C and Figure 3B,D). The expression of the epithelial marker genes WT1 and CDH1 was similarly affected in primary EPDCs of PD5 as in iEPDCs of PD15 and -30 (WT1) and of PD15 (CDH1). Five days after SB removal, all cells displayed an increase in expression of the mesenchymal marker genes CDH2, ACTA2, COL1A1, and FN1 (Figure 2C and Figure 3B,D). Culturing of the cells for 5 days in the presence of TGFβ3, led in most cases to a strong further increase in mesenchymal marker gene expression (Figure 2C and Figure 3B,D). Comparison of both epithelial and mesenchymal marker genes between primary EPDCs of PD5 and iEPDCs of PD25, -28, -30 and -35 (corresponding to four biological replicates) demonstrated similar gene expression levels and displayed similar changes in expression pattern after removal of SB or addition of TGFβ for most of the genes. However, CDH1 in particular was expressed at lower levels in iEPDCs when compared to primary EPDCs under all conditions. (Supplemental Figure S3A,B). Expression of epithelial and mesenchymal markers was confirmed at the protein level by Western blotting. As shown in Supplemental Figure S3B, iEPDCs and primary EPDCs displayed very similar changes in the levels of E-cadherin, N-cadherin, α-smooth muscle actin, and fibronectin upon induction of EMT.

### 3.3. Proliferating iEPDCs Show a Reduced Propensity to Undergo EMT upon TGFβ3 Stimulation

As mentioned previously, TGFβ can induce EMT in EPDCs while inhibition of TGFβ signaling can prevent EMT of these cells. Interestingly, in in vitro cultures we observed a PD-dependent propensity for primary human EPDCs to undergo EMT. This change in phenotype could not be prevented by TGFβ-ALK5 pathway inhibition and was often accompanied by a decreased proliferation rate of the cells.

**Figure 4:**
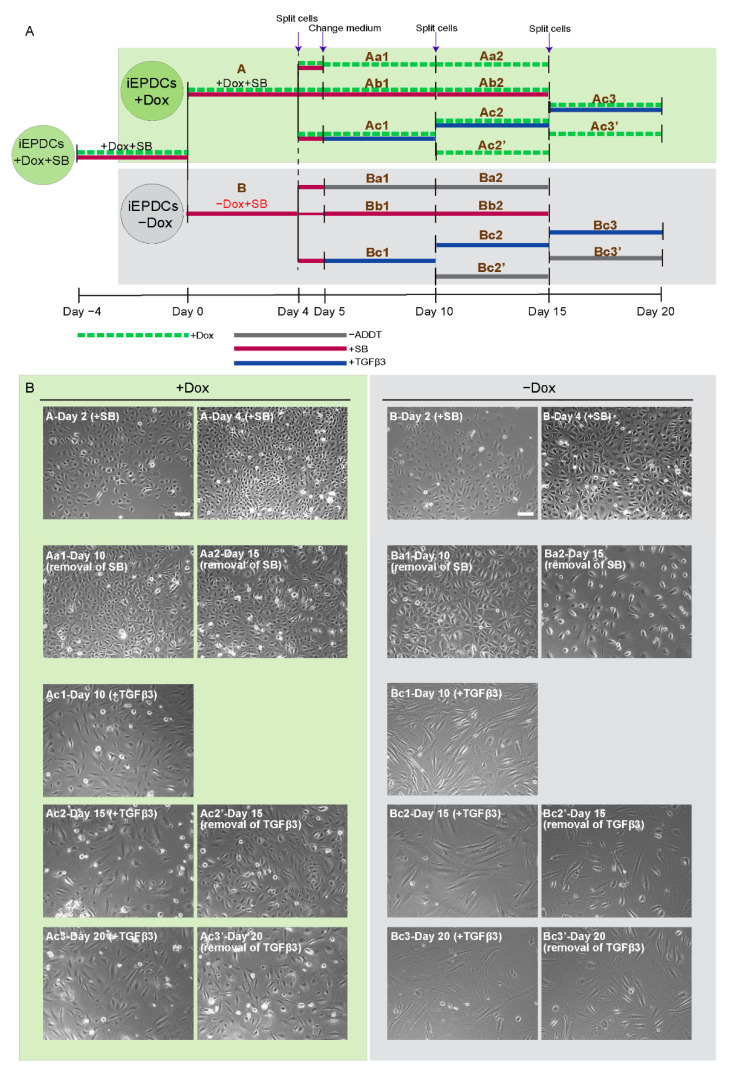
Proliferating iEPDCs show a reduced propensity to undergo EMT upon TGFβ3 stimulation. (**A**). Diagram of the experimental protocol used to examine EMT of actively proliferating (+Dox) and cell cycle-arrested (−Dox) iEPDCs following exposure to TGFβ3 (1 ng/mL) or various control conditions. (**B**). Representative phase contrast images of iEPDCs subjected to the treatment regimens shown in (**A**). The images on the left panel display the morphology of actively proliferating iEPDCs under each condition, the images in the right panel show the morphology of cell cycle-arrested iEPDCs under each condition. While TGFβ3 treatment readily induces EMT in non-dividing iEPDCs, dividing cells only displayed signs of EMT after prolonged exposure to TGFβ3. Scale bar = 100 µm. −ADDT = no additive.

Therefore, we tested the response of iEPDCs to TGFβ treatment in the presence of Dox to maintain a high proliferation rate and in its absence to induce cell cycle arrest. As depicted in Figure 4A, iEPDCs cultured in the presence or absence of Dox (designated iEPDCs + Dox and iEPDCs − Dox, respectively) were exposed to complete medium containing SB, TGFβ, or neither of the two (control conditions) for the indicated times after which cells were judged morphologically for their tendency to undergo EMT.

After four days of culture in the absence of Dox and presence of SB, iEPDCs stopped proliferating and had a similar morphology to primary EPDCs cultured in SB-containing medium (Figure 4B, panels B-Day 2 (+SB) and B-Day 4 (+SB), Supplemental Figure S4). iEPDCs that were cultured for four days in the presence of Dox and with SB continued to divide (compare Figure 4B, panel A-Day 2 (+SB) with A-Day 4 (+SB)) and did not undergo EMT. Subsequent removal of SB from the culture medium did not induce EMT in iEPDCs + Dox or iEPDCs − Dox based on morphological observations (Figure 4B, panels Aa1&2 and Ba1&2, Supplemental Figure S4). However, iEPDCs + Dox and iEPDCs − Dox showed markedly different responses to TGFβ3 stimulation. Compared to the typical transformation of iEPDCs − Dox after five days of stimulation with TGFβ3 into spindle-shaped mesenchymal EPDCs, the iEPDCs + Dox were not fully transformed upon TGFβ3 treatment (compare Figure 4B, panel Bc1 with Ac1). Continued culture in medium with or without TGFβ3 did not induce additional changes in the cell morphology of iEPDCs − Dox (Figure 4B, panels Bc2&3 and Bc2′&3′). After prolonged TGFβ3 stimulation (up to 15 days), iEPDCs + Dox still did not acquire a typical mesenchymal morphology (Figure 4B, panels Ac2 and Ac3). Furthermore, when removing TGFβ3 after 10 days of stimulation, iEPDCs + Dox reacquired a cobblestone-like appearance (Figure 4B, panels Ac2′and Ac3′).

### 3.4. Mesenchymal iEPDCs Show Robust Migration and Invasion Ability

Critical to their participation in heart development and cardiac regeneration, is the ability of a subset of epicardial cells to (i) undergo EMT, (ii) acquire migration capacity, and (iii) invade the subepicardial space and myocardium to subsequently differentiate into cardiac fibroblasts and vascular smooth muscle cells [34,35,36].

**Figure 5:**
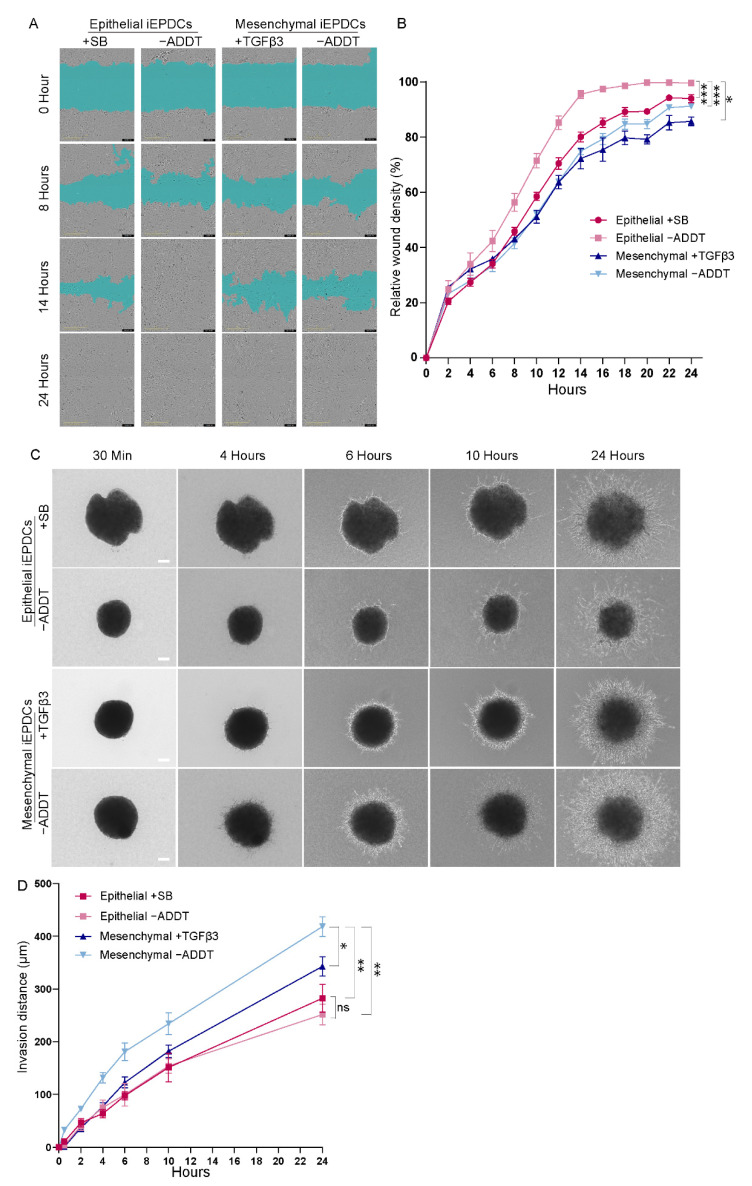
Mesenchymal iEPDCs show robust migration and invasion ability (**A**) Representative phase contract images of iEPDC migration in a scratch wound healing assay. Scale bar = 400 µm. (**B**). Quantification of iEPDC migration in a scratch wound healing assay. (**C**). Representative images of iEPDC aggregates in a 3D collagen gel-based invasion assay. Scale bar = 100 µm. (**D**). Quantification of EPDC invasion distance within 24 h. The assay was performed in Dox-free complete medium with the indicated additives using either epithelial iEPDCs (i.e., iEPDCs pretreated with SB (10 µM) to preserve their cuboidal epithelial morphology) or with mesenchymal iEPDCs (i.e., iEPDCs pretreated for five days with TGFβ3 (1 ng/mL) to induce a spindle-like morphology). * *p* < 0.05, ** *p* < 0.01, *** *p* < 0.001. −ADDT = no additive.

To compare the migration ability of non-proliferating epithelial iEPDCs with that of non-proliferating mesenchymal iEPDCs, a classical scratch wound healing assay was performed. As shown in Figure 5A, both epithelial and mesenchymal iEPDCs (gray area) migrate into the cell-free zone (cyan area) and gradually close the wound. Although the difference was rather small, wound closure occurred faster in the epithelial iEPDC cultures compared to the mesenchymal iEPDC cultures. Quantification of the wound closure rate showed it to be fastest for epithelial iEPDCs cultured without SB during the assay (Figure 5B), which is consistent with our previous findings in primary human EPDCs [12]. Analyzing the cell migration patterns in more detail, we found that epithelial iEPDCs closed the wound by migrating as a cell sheet, whereas mesenchymal iEPDCs tended to migrate individually into the cell-free zone (Figure 5A, Supplemental videos). The migration speed of mesenchymal iEPDCs based on the quantification of relative wound density may therefore be underestimated, especially in the beginning of the assay because individual cell migration cannot be recognized as easily as cell sheet-like migration. The specific migration pattern of epithelial iEPDCs observed in the scratch wound healing assay is reminiscent of the behavior of epicardial cells during cardiac development, i.e., gradually covering the whole heart as a cell sheet.

Another key function of EPDCs is their ability to invade the underlying myocardium after EMT. We employed a 3D invasion assay in order to further investigate this function of epithelial and mesenchymal iEPDCs. As can be appreciated in Figure 5C, iEPDCs gradually migrate out of cell aggregates and invade the surrounding collagen gel, moving further away from the aggregates. Mesenchymal iEPDCs cultured in the absence of TGFβ3 during the assay spread through the collagen gel significantly faster than epithelial iEPDCs or TGFβ3-stimulated mesenchymal iEPDCs (Figure 5D). No difference in cell migration was observed between epithelial iEPDCs that were cultured in medium with or without SB during the time of this assay, indicating that inhibition of TGFβ-ALK5 signaling did not affect epithelial EPDCs’ invasion capacity. The presence of TGFβ3 during the assay decreased the cell invasion ability of the mesenchymal EPDCs, which may be the result of the continuing differentiation of the mesenchymal EPDCs by lasting TGFβ stimulation. TGFβ is known to transiently induce EMT in vivo during development, and both in vitro and in vivo studies have shown that TGFβ signaling promotes EPDC differentiation and their contribution to heart development and regeneration [13,37,38]. The phenotype (epithelial or mesenchymal)- and TGFβ-ALK5 signaling (with or without SB or TGFβ3)-dependent changes in invasion capacity of iEPDCs mimicked those observed in primary adult human EPDCs (Supplemental Figure S5). Interestingly, both mesenchymal and epithelial iEPDCs displayed a much higher capacity to invade collagen gels in comparison to their primary counterparts. To investigate whether this might be linked to the lower CDH1 expression in iEPDCs (Supplemental Figure S3), the cells were transduced with an LV encoding human E-cadherin. After confirmation of E-cadherin overexpression by immunofluorescence microscopy (Supplemental Figure S6A), the invasion capacity of epithelial and mesenchymal subtypes of these iEPDCs was investigated in the 3D invasion assay. As shown in Supplemental Figure S6B, E-cadherin overexpression only slightly reduced the invasion capacity of both epithelial and mesenchymal iEPDCs.

### 3.5. Mesenchymal iEPDCs Promote Neurite Outgrowth from Sympathetic Ganglia

**Figure 6:**
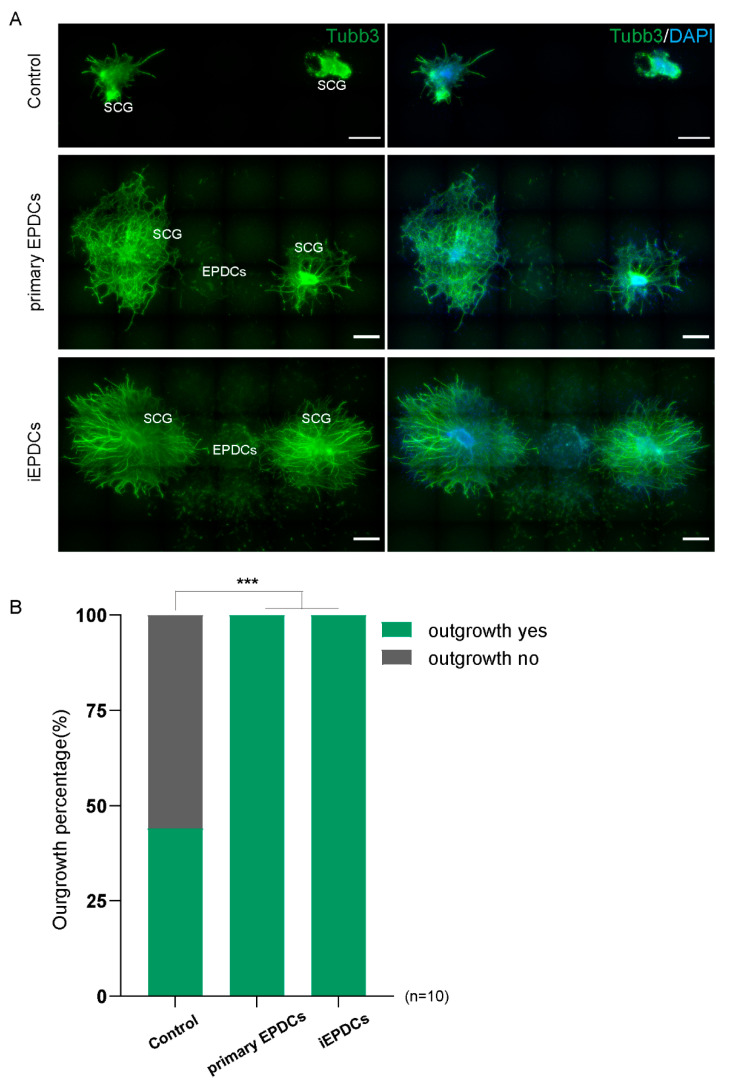
Mesenchymal iEPDCs promote neurite outgrowth of sympathetic ganglia. (**A**). Representative immunofluorescence images (Tubb3 staining) of embryonic superior cervical ganglia (SCG) outgrowth after six-day culture alone or together with primary human EPDCs or iEPDCs in a 3D culture setup. Upper panel: No or hardly any neurite outgrowth is observed when SCG are cultured without EPDCs. Co-culture with primary human EPDCs (PD8, middle panel) or iEPDCs (PD30, lower panel) promotes neurite outgrowth from SCG. (**B**). Quantification of the percentage of SCGs showing neurite outgrowth when cultured alone or co-cultured with primary human EPDCs or iEPDCs. Scale bar = 500 µm. *** *p* < 0.001.

We recently showed that primary adult human mesenchymal EPDCs promote neurite outgrowth from sympathetic ganglia in vitro by a paracrine effect [16]. To study whether the same results could be obtained using iEPDCs, primary EPDCs and iEPDCs derived from the same patient were treated with TGFβ3 for five days to induce EMT and passaged twice in Dox-free medium without TGFβ3. Next, the EPDCs were co-cultured with superior cervical ganglion (SCG) explants for six days without additive. Consistent with our previous findings, less than 50% of the SCG explants showed limited outgrowth when cultured without EPDCs (Figure 6A,B) [16]. In contrast, co-culture of SCG with both mesenchymal primary EPDCs and with iEPDCs robustly stimulated ganglionic outgrowth (Figure 6A,B).

## 4. Discussion

The interest in the epicardium has strongly increased during recent decades due to its (i) key role in heart development, (ii) involvement in cardiac injury responses, and (iii) potential to contribute to myocardial regeneration both through paracrine signaling and by differentiation of its cellular descendants into cardiac fibroblasts and vascular smooth muscle cells [10]. To facilitate human EPDC research, our research teams previously developed methods to isolate and culture primary human epicardial cells for in vitro studies and xenogeneic transplantation experiments [11,12,14,17]. Further characterization of EPDCs to better understand their contribution to myocardial fibrosis and to fully exploit their cardioregenerative potential is, however, hampered by (i) the limited availability of human epicardium and EPDCs for research purposes, (ii) the highly variable properties of EDPCs obtained from different donors, and (iii) the fact that primary EPDCs stop dividing and lose their epithelial phenotype after a few passages in vitro. The availability of a well-characterized line of human EPDCs phenotypically resembling primary human EPDCs would greatly expedite epicardial research and allow high-throughput studies, e.g., to identify (novel) drugs that can modulate EPDC function. Here, we introduce the first adult human epicardial cell line with Dox-inducible proliferation capacity via expression of SV40 LT using a repressor-based Tet-On system. This human epicardial cell line recapitulates key properties of primary epicardial cells and overcomes important challenges that come with the use of primary EPDCs.

There are various options to extend the proliferative capacity of primary mammalian cells [39,40]. Viral oncoproteins of DNA tumor viruses (in particular the SV40 LT + small t antigen [st], the human papillomavirus type 16/18 E6 and E7 proteins and the human adenovirus type 2/5 E1A and E1B proteins) have been extensively used for this purpose [41]. This has, amongst other things, resulted in the generation of multiple lines of human endothelial cells [42], hepatocytes [43], lung epithelial cells [44], and keratinocytes [45]. The first lines of human mesothelial cells were published in 1989 [46] and were generated by transfection of cells isolated from pleural fluid with a plasmid encoding the early gene products (i.e., LT, 17kT and st) of SV40. This extended the replicative life span of the cells from 15 PDs to up to 60–70 PDs and gave rise to a single clone of permanently immortalized mesothelial cells with a cuboidal epithelial morphology designated MeT-5A. Subsequently, several other lines of human pleural and peritoneal mesothelial cells were generated (see, e.g., Fischereder et al. 1997 [47]; Pruett et al. 2020 [48]). However, the present paper is the first to describe the generation of a human epicardial mesothelial cell line.

In the mesothelial cell lines generated by Ke et al. [46] and Fischereder et al. [47], LT expression is under the control of constitutively active promoters. For many cell types, continuous LT expression/cell proliferation negatively affects their functional properties. We therefore employed a repressor-based lentiviral Tet-On system allowing tight control of LT expression by Dox to generate the iEPDCs described in this study. We have previously used very similar conditional cell immortalization systems to generate lines of neonatal rat atrial cardiomyocytes with preserved cardiomyogenic differentiation capacity [22] and for producing adipogenic lines of murine and human brown preadipocytes (designated iBPAs) [21]. The latter study nicely illustrated the advantage of inducible over permanent immortalization gene expression, as the human iBPAs could only differentiate into fat cells in the absence of Dox, i.e., after switching off LT expression. In congruence with these findings, iEPDCs displayed a reduced ability to undergo TGFβ3-induced EMT in the presence of Dox.

For the application of iEPDCs, it is mandatory that they possess similar characteristics to primary human EPDCs with regard to morphology, molecular signature, and function. Morphologically, early passage SB-treated primary human EPDCs strongly resemble SB-treated iEPDCs and both cell types undergo EMT adapting a spindle-like appearance after exposure to TGFβ3. Moreover, comparative gene expression analysis between primary human EPDCs and iEPDCs showed that the epithelial marker genes WT1, BNC1, and ALDH1A2 were expressed at very similar levels in both cell types and that mesenchymal marker gene expression was affected similarly upon treatment of primary human EPDCs and iEPDCs with EMT modulators. One exception was the epithelial marker gene CDH1, which showed a lower expression in iEPDCs as compared to primary human EPDCs under all experimental conditions. CDH1 codes for E-cadherin, which is responsible for the tight cell–cell contact in adherent junctions connecting neighboring EPDCs. During EMT, expression of junctional proteins including E-cadherin is lost, which results in the disruption of intercellular junctions and together with other changes in cell behavior, allows EPDCs to migrate into the myocardium [10,49]. Consistent with their decreased E-cadherin expression, iEPDCs displayed a higher invasion capacity in 3D collagen hydrogels than primary human EPDCs. While CDH1 overexpression reduced the invasion ability of iEPDCs, it remained higher than that of primary human EPDCs, indicating that additional factors contribute to the increased motility of iEPDCs. Although loss of E-cadherin has been associated with the initiation of EMT, non-TGFβ3-stimulated iEPDCs maintained a cuboidal epithelial phenotype, suggesting that their E-cadherin levels are high enough to prevent EMT.

The iEPDCs started to lose their cuboidal epithelial appearance at PD51, which was accompanied by a slowing of the cell division rate. It will be of interest to determine the reason(s) for this change in cell morphology and doubling time. Conceivably, iEPDCs gradually accumulate telomeric and non-telomeric DNA damage due to exposure to the high atmospheric O_2_ level and/or to the high replication rate imposed by LT, which, in combination with telomere shortening, result in the induction of cellular senescence. If so, combined overexpression of LT and telomerase reverse transcriptase and measures to reduce DNA damage during in vitro culture might further expand the replicative life span of epithelial iEPDCs, especially when derived from older patients. Alternatively, the changes in cell morphology observed at late passages of the iEPDCs are caused by the progressive loss of the epigenetic determinants of their epithelial cell identity [50]. To prevent this from happening will require the identification of the epigenetic memory initiating factors responsible for the maintenance of the cuboidal epithelial appearance of EPDCs [51].

The fact that proliferation of the iEPDCs is strictly Dox-dependent allowed us to investigate the relationship between proliferation and EMT of EPDCs. As mentioned above, continuous proliferation induced by Dox inhibited the ability of iEPDCs to undergo EMT in response to TGFβ3 stimulation. Moreover, iEPDCs cultured in the presence of Dox reacquired epithelial characteristics when exposure to TGFβ3 was stopped. This is in line with the previous description of competition between cell growth and differentiation in normal, genetically unmodified somatic (stem) cells [52,53,54] and may suggest that EMT and proliferation are competing processes in iEPDCs, where high proliferation is accompanied by low EMT, and vice versa. This finding is consistent with our observation that primary human EPDCs spontaneously undergo EMT in the absence of TGFβ3 after entering a low proliferation state in vitro. Studies in other cell types support the existence of a possible relationship between cell proliferation and EMT, e.g., in tumor cells EMT has been shown to inhibit cell proliferation whereas inhibition of EMT promotes tumor cell proliferation [55,56]. However, since SV40 can induce malignant mesotheliomas in rodents [57] and this effect is linked to expression of LT, caution should be exercised in drawing definitive conclusions about the relationship between cell proliferation and EMT in EPDCs due to possible direct effects of LT on EMT.

Taken together, in this study we have shown that human EPDCs can be endowed with inducible proliferation capacity and massively expanded without loss of their cuboidal epithelial morphology and characteristic (functional) properties by Dox-controlled expression of SV40 LT using a lentiviral repressor-based Tet-On system. Similar to primary human EPDCs, iEPDCs underwent EMT upon TGFβ3 stimulation as evidenced by the acquisition of a spindle-shaped appearance and by the upregulation of mesenchymal marker genes. Moreover, functional collagen gel-based cell invasion assays demonstrated that mesenchymal iEPDCs, just like primary human EPDCs had an increased capacity for invasion and migration as compared to their epithelial counterparts. Finally, mesenchymal iEPDCs were shown to stimulate neurite outgrowth from sympathetic ganglia by paracrine signaling similar to primary human EPDCs [16]. In conclusion, iEPDCs provide a plentiful cell source for fundamental and translation EPDC research and as such represent a valuable new addition to the existing epicardial model systems.

## Data Availability

Not applicable.

## Supplementary Materials

The following are available online at https://www.mdpi.com/article/10.3390/cells10082064/s1, Supplemental material and methods. Figure S1: Schematic overview of iEPDC generation, Figure S2. Passaging of primary human EPDCs results in loss of their epithelial cuboidal morphology, Figure S3. Assessment by RT-qPCR and western blotting of epithelial and mesenchymal marker gene expression in primary human EPDCs and in iEPDCs, Figure S4. Appearance of iEPDCs after long-term culture in the presence of SB with or without Dox, Figure S5. Invasion ability of epithelial and mesenchymal primary human EPDCs, Figure S6. CDH1 overexpression reduces the invasion ability of mesenchymal EPDCs, Table S1: qPCR primers, Table S2: Western blot reagents, Video S1: Epithelial iEPDC (+SB) migration, Video S2: Epithelial iEPDC (−ADDT) migration, Video S3: Mesenchymal iEPDCs (+TGFβ3) migration, Video S4: Mesenchymal iEPDCs (−ADDT) migration.
