# Supplementary material for: Generation, Characterization, and Application of Inducible Proliferative Adult Human Epicardium-Derived Cells
Source: Cells. 2021 Aug 12;10(8):2064. doi: 10.3390/cells10082064 (PMC8391799; doi:10.3390/cells10082064)
Supplement: Supplementary file 1 [file cells-10-02064-s001.zip › Supplemental materials.pdf]

Supplemental Methods

CDH1 Overexpression in iEPDCs

Overexpression of human E-cadherin (CDH1) in iEPDCs was accomplished with an LV containing a bicistronic expression unit consisting of the human eukaryotic translation elongation factor 1α gene promoter, the coding sequence of human CDH1, an encephalomyocarditis virus internal ribosomal entry site and the coding sequence of the *Aequorea victoria* enhanced green fluorescent protein. The shuttle plasmid for making this LV was obtained from Addgene (Watertown, MA; pHAGE-CDH1; plasmid number 116722). The production, purification and concentration of LV particles was done essentially as described in [1].

Senescence β-Galactosidase Staining

To investigate senescence in iEPDCs at PD18, -29 and -50, a Senescence β-Galactosidase Staining Kit (#9860; Cell Signaling Technology, Leiden, the Netherlands) was applied as recommended by the manufacturer. Imging acquisition was done with the EVOS FL Auto 2 Imaging System (Thermo Fisher Scientific).

Western Blotting

Eight days after removal of Dox, iEPDCs at PD23, -30 and -34) and primary EPDCs were cultured for 5 days in complete medium containing SB (10 μM), no additive or TGFβ3 (1 ng/ml). Next, cells were lysed in ice-cold RIPA Lysis and Extraction Buffer supplemented with Halt Protease Inhibitor Cocktail and the lysates were passed ≥3 times through a 30G needle and centrifuged at 16000×*g* for 20 min at 4°C. The total protein concentration in each sample was determined with the Pierce BCA Protein Assay Kit and 13 μg of each protein sample was subjected to polyacrylamide gel electrophoresis for subsequent blotting. For fibronectin and E-cadherin, a NuPAGE 3-8% Tris-Acetate Gel and corresponding running buffer were used for protein fractionation and lamin A/C served as loading control. For N-cadherin and α-smooth muscle actin, a Bolt 10% Bis-Tris Plus Gel and corresponding running buffer were used and glyceraldehyde 3-phosphate dehydrogenase (GAPDH) served as loading control. After electrophoresis, size-fractionated proteins were transferred to 0.45-μm polyvinylidene difluoride membranes (GE Healthcare, Chicago, IL) by wet electroblotting. Next, membranes were incubated for 1 hour in 2% ECL Prime Blocking Reagent in Tris-based saline/0.1% Tween-20 (TBST). Membranes were then incubated overnight at 4°C with primary antibodies in TBST/2% ECL Prime Blocking Reagent, washed 3 times with TBST and incubated for 1 hour with matching horseradish peroxidase-conjugated secondary antibodies. Following 3 washes with TBST, the membranes were incubated with SuperSignal West Femto Maximum Sensitivity Substrate and chemiluminescence was measured using the iBright FL1500 Imaging System (Thermo Fisher Scientific). After detection of the proteins of interest, the blots were stripped and immunostained for the loading controls lamin A/C and GAPDH. Details about the different reagents used for western blotting are provided in **Supplemental Table 2**.

E-cadherin Immunostaining

iEPDCs were fixed with 4% paraformaldehyde in PBS, permeabilized with 0.5% Tween 20 (822184; Merck Millipore, Darmstadt, Germany) in PBS and non-specific epitopes were blocked with PBS containing 1% bovine serum albumin (A8022; Sigma-Aldrich, St. Louis, MO) and 0.05% Tween 20. Afterwards, iEPDCs were stained with rabbit anti-E-cadherin antibodies (Abcam, Cambridge, MA; ab40772; 1:500) overnight at 4°C. After 3 washes with 0.05% Tween in PBS, the cells were incubated with Alexa Fluor 647-conjugated donkey anti-rabbit IgG(H+L) (Thermo Fisher Scientific, Bleiswijk, the Netherlands A-31573; 1:250) and Alexa Fluor 594-conjugated phalloidin (Thermo Fisher Scientific A12381, 1:200) at room temperature for 1 hour. DAPI (300 nM; D3571; Thermo Fisher Scientific) was used to stain nuclei. All images were captured with a Leica TCS SP8 confocal laser scanning microscope (Leica Microsystems, Wetzlar, Germany).

Invasion Assay of CDH1-Overexpressing iEPDCs

Eight days after removal of Dox, the iEPDCs were cultured for 5 days in complete medium containing SB or TGFβ3 to keep epithelial iEPDCs or obtain mesenchymal iEPDCs, respectively. Next, aggregates of 20,000 epithelial iEDPCs, mesenchymal iEPDCs or primary EPDCs in a total volume of 30 μl were formed by the hanging drop technique. Twenty four hours before the invasion assay started, the epithelial iEPDCs in the hanging drop were given complete medium with or without SB while the clumps of mesenchymal EPDCs received complete medium with or without TGFβ3. To measure their invasion ability, aggregates of iEPDCs that did or did not overexpress CDH1 were placed in drops of 3 mg/ml rat tail collagen I (354236; Corning Life Sciences, Amsterdam, the Netherlands) and cultured for the indicated time periods in EPDC culture medium. Time-lapse images were captured immediately with the EVOS FL Auto 2 Imaging System. The invasion distance of the cells away from the aggregates were measured with ImageJ 1.52p (National Institutes of Health [NIH], Bethesda, MA).

Supplemental Table S1. qPCR primers.

Gene		Sequence
**GAPDH**	Forward	AGCCACATCGCTCAGACAC
	Reverse	GCCCAATACGACCAAATCC
**TBP**	Forward	TGGAAAAGTTGTATTAACAGGTGCT
	Reverse	GCAAGGGTACATGAGAGCCA
**HPRT1**	Forward	CTCATGGACTGATTATGGACAGGAC
	Reverse	GCAGGTCAGCAAAGAACTTATAGCC
**WT1**	Forward	CAGCTTGAATGCATGACCTG
	Reverse	TATTCTGTATTGGGCTCCGC
**BNC1**	Forward	CCACCGTCAGTGTGACCAAT
	Reverse	CAATCTCCACCTGGCTTGTT
**ALDH1A2**	Forward	AACAAGGCCCTCACAGTGTC
	Reverse	TTCTGAGTACTCCCGCAAGC
**CDH1**	Forward	CCCGGTATCTTCCCCGC
	Reverse	CAGCCGCTTTCAGATTTTCAT
**CDH2**	Forward	CAGACCGACCCAAACAGCAAC
	Reverse	GCAGCAACAGTAAGGACAAACATC
**ACTA2**	Forward	CCGGGAGAAAATGACTCAAA
	Reverse	GAAGGAATAGCCACGCTCAG
**COL1A1**	Forward	CAGGCTGGTGTGATGGGATT
	Reverse	GGGCCTTGTTCACCTCTCTC
**FN1**	Forward	CGTCATAGTGGAGGCACTGA
	Reverse	CAGACATTCGTTCCCACTCA

Supplemental Table S2. Western blot reagents.

Device and Reagent	
**RIPA Lysis and Extraction Buffer**	89900; Thermo Fisher Scientific
**Halt™ Protease Inhibitor Cocktail**	87785; Thermo Fisher Scientific
**Pierce BCA Protein Assay Kit**	23227; Thermo Fisher Scientific
**NuPAGE™ 3-8% Tris-Acetate Gel**	EA03785BOX; Thermo Fisher Scientific
**NuPAGE™ Tris-Acetate SDS Running Buffer (20X)**	LA0041; Thermo Fisher Scientific
**NuPAGE™ Transfer Buffer (20X)**	NP00061; Thermo Fisher Scientific
**Bolt™ 10% Bis-Tris Gel**	NW00105BOX; Thermo Fisher Scientific
**Bolt™ MOPS SDS Running Buffer (20X)**	B000102; Thermo Fisher Scientific
**Bolt™ Transfer Buffer (20X)**	BT0006; Thermo Fisher Scientific
**Mini Gel Tank and Blot Module Set**	NW2000; Thermo Fisher Scientific
**ECL Prime blocking reagent**	RPN418V; Sigma-Aldrich
**SuperSignal™ West Femto Maximum Sensitivity Substrate**	34094; Thermo Fisher Scientific
**Western Blot Stripping Buffer**	21059; Thermo Fisher Scientific
**iBright FL1500 Imaging System**	Thermo Fisher Scientific
Antibody	
**Mouse-anti-fibronectin**	1:500; F6140; Sigma-Aldrich
**Rabbit-anti-E-cadherin**	1:5000; ab40772; Abcam
**Mouse-anti-N-cadherin**	1:200; C3865; Sigma-Aldrich
**Rabbit-anti-α-smooth muscle actin (αSMA)**	1:200; ab5694; Abcam
**Rabbit-anti-Lamin A/C**	1:5000; SC-20681; Santa Cruz
**Mouse-anti-glyceraldehyde 3-phosphate dehydrogenase (GAPDH)**	1:300; MAB374; Merck (Millipore)
**HRP~Goat-anti-Mouse IgG(H&L)**	1:25000; ab97040; Abcam
**HRP~Goat-anti-Rabbit IgG(H&L)**	1:25000; ab97080; Abcam

Reference

1. Liu, J.; Volkers, L.; Jangsangthong, W.; Bart, C.I.; Engels, M.C.; Zhou, G.; Schalij, M.J.; Ypey, D.L.; Pijnappels, D.A.; de Vries, A.A.F. Generation and primary characterization of iAM-1, a versatile new line of conditionally immortalized atrial myocytes with preserved cardiomyogenic differentiation capacity. *Cardiovasc. Res.* **2018**, *114*, 1848–1859, doi:10.1093/cvr/cvy134.

Supplemental Figures

Supplemental Figure S1

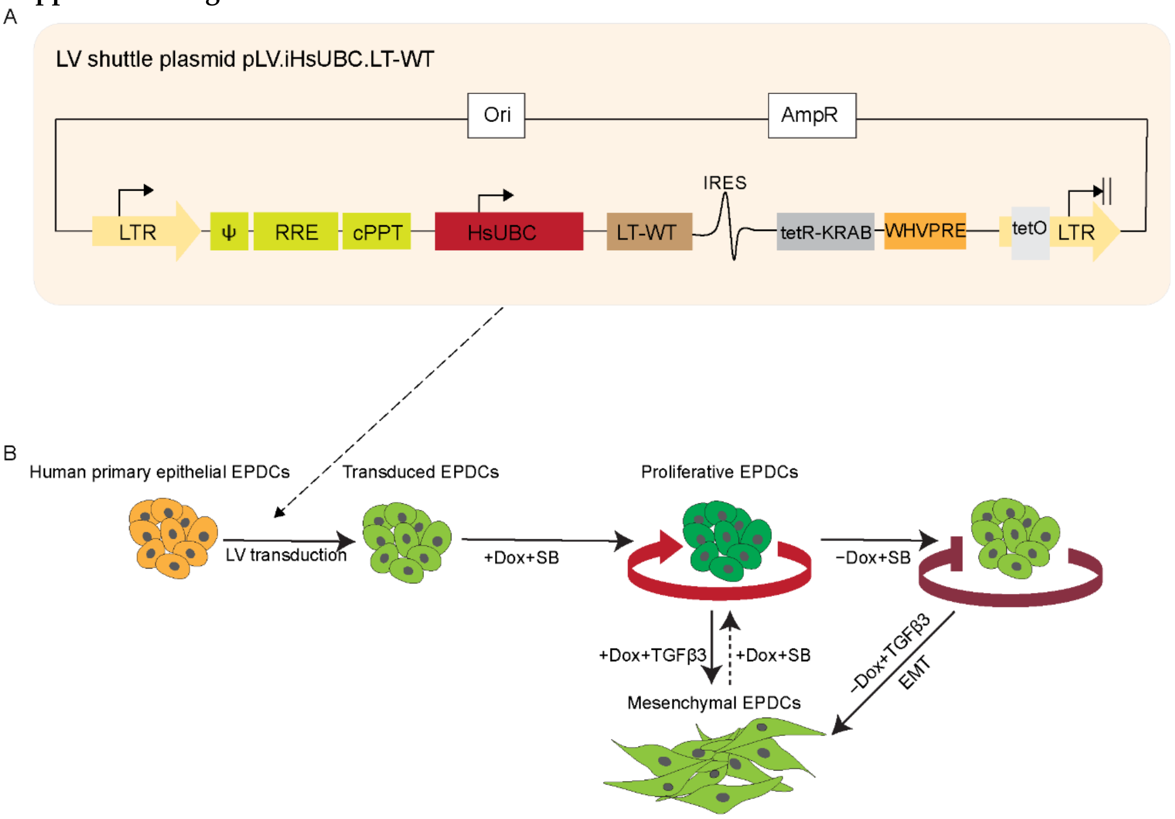

Supplemental Figure S1. Schematic overview of iEPDC generation. (**A**) Map of the LV shuttle plasmid to generate LV.iHsUBC.LT-WT. LTR, human immunodeficiency virus type 1 (HIV1) long terminal repeat; Ψ, HIV1 packaging signal; RRE, HIV1 Rev-responsive element; cPPT, HIV1 central polypurine tract and termination site; HsUBC, human ubiquitin C gene promoter; LT-WT, coding sequence of the wildtype SV40 LT protein, IRES, encephalomyocarditis virus internal ribosome entry site; tetR-KRAB, coding sequence of the hybrid tetracycline-controlled transcriptional repressor; WHVPRE, woodchuck hepatitis virus posttranscriptional regulatory element; tetO, tetracycline-responsive promoter element consisting of 7 repeats of a 19-nucleotide tetracycline operator sequence; AmpR, *Escherichia coli* ß-lactamase gene; Ori, bacterial origin of replication (**B**) Diagram showing the derivation of epithelial and mesenchymal iEPDCs.

Supplemental Figure S2

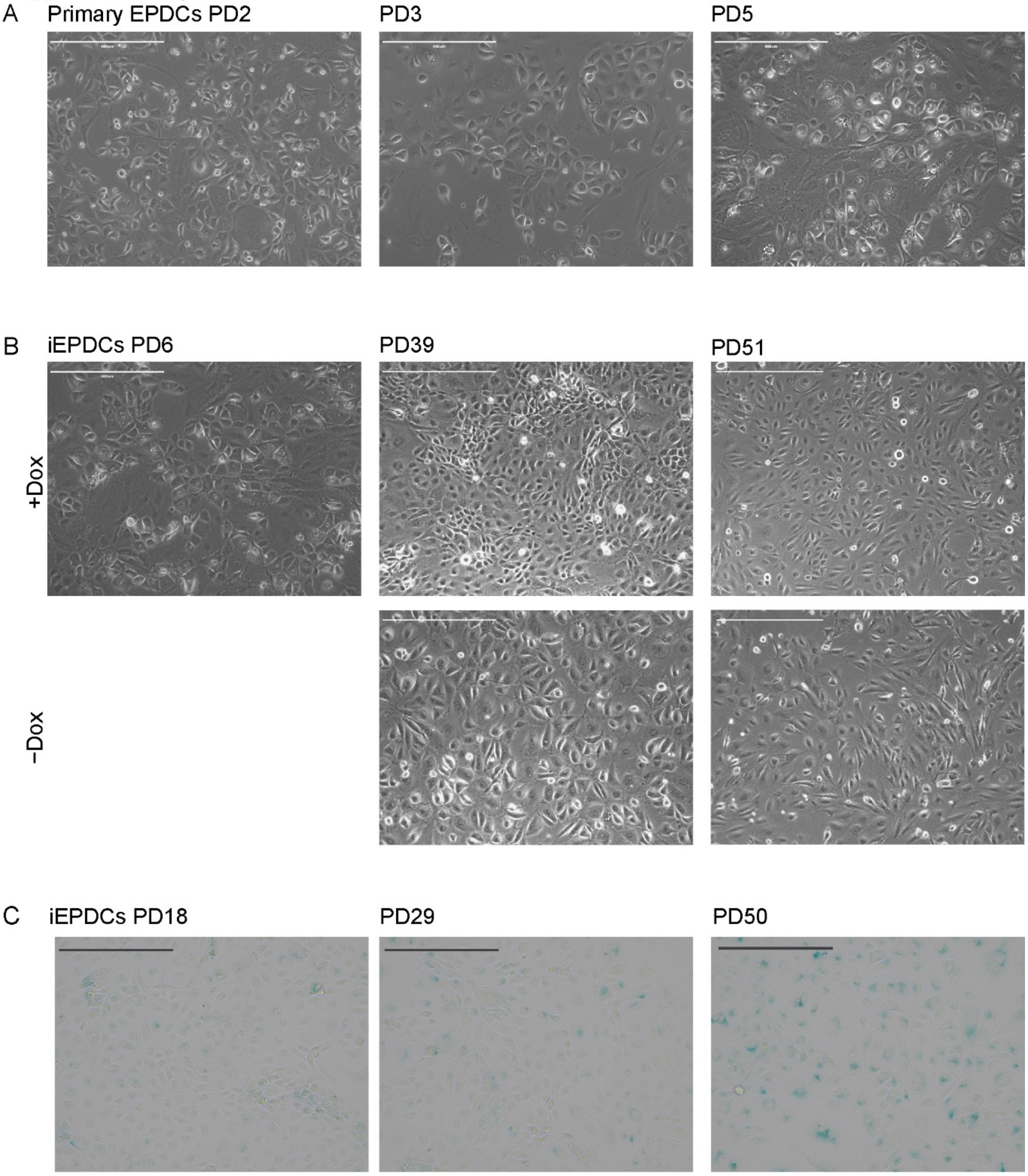

Supplemental Figure S2. Passaging of primary human EPDCs results in loss of their epithelial cuboidal morphology. (**A**) Representative phase contrast images of primary human EPDCs of PD2, -3 and -5 showing loss of cuboidal epithelial morphology by a fraction of the cells at PD5. (**B**) Representative phase contrast images of iEPDCs of PD6, -39 and -51. iEPDCs still have a cuboidal epithelial morphology at PD39 but start to display a more spindle-like appearance at PD51. (C) Bright field images of senescence β-galactosidase staining of iEPDCs at PD18, -29 and -50. Scale bar = 400 μm.

Supplemental Figure S3

A

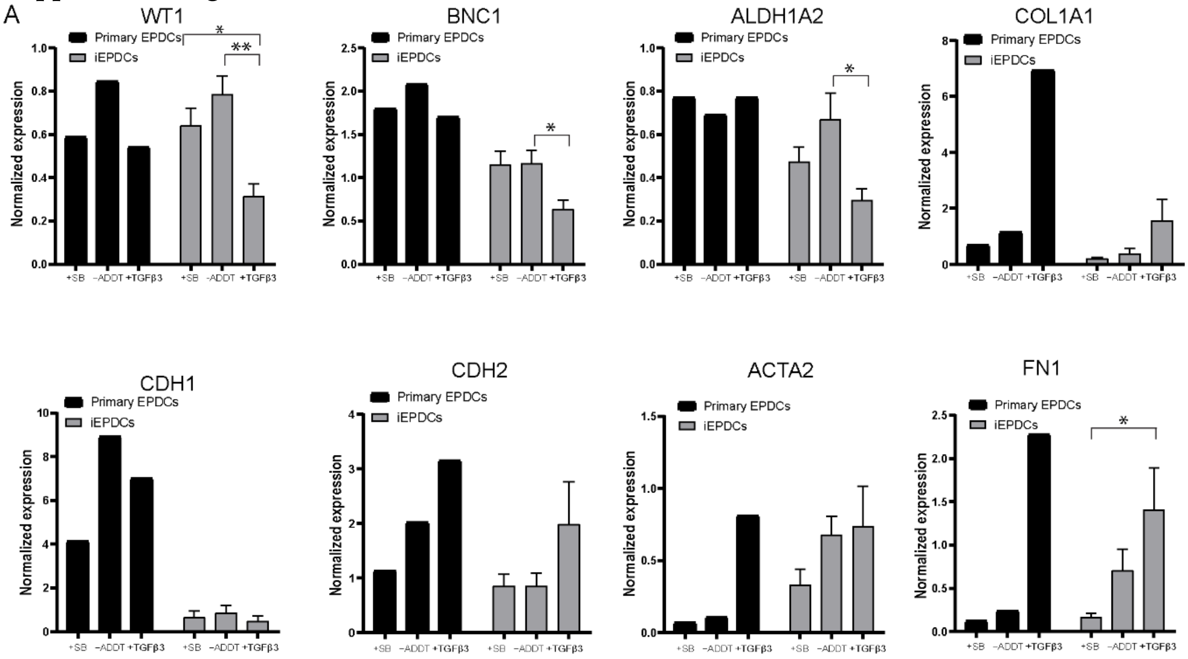

B

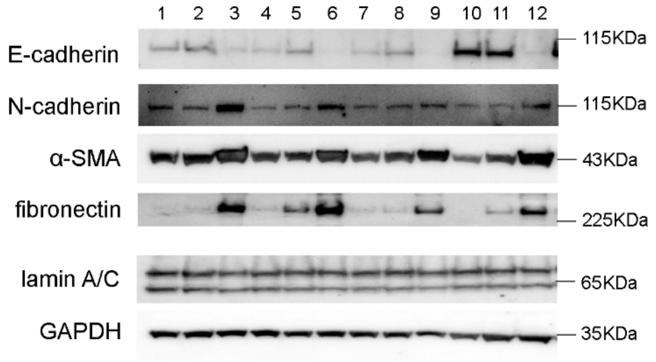

Lane	
1	iEPDCs PD23 +SB
2	iEPDCs PD23 −ADDT
3	iEPDCs PD23 +TGFβ3
4	iEPDCs PD30 +SB
5	iEPDCs PD30 −ADDT
6	iEPDCs PD30 +TGFβ3
7	iEPDCs PD34 +SB
8	iEPDCs PD34 −ADDT
9	iEPDCs PD34 +TGFβ3
10	Primary EPDCs PD4 +SB
11	Primary EPDCs PD4 −ADDT
12	Primary EPDCs PD4 +TGFβ3

Supplemental Figure S3. Assessment by RT-qPCR and western blotting of epithelial and mesenchymal marker gene expression in primary human EPDCs and in iEPDCs. (**A**) RT-qPCR analysis. Black colored bars represent primary human EPDCs of PD5. Gray colored bars represent cumulative data of iEPDCs of PD25, -28, -30 and -35. For each sample, 3 technical replicates were performed. Data is shown as mean±SEM. *P<0.05, **P<0.01. (**B**) Western blot analysis of primary EPDCs and of iEPDCs at PD23, -30 and -34 for E-cadherin, N-cadherin, α-smooth muscle actin (α-SMA) and fibronectin. iEPDCs and primary EPDCs were cultured without Dox under conditions that either inhibited (+SB) or stimulated (+TGFβ3) EMT. Lamin A/C and glyceraldehyde 3-phosphate dehydrogenase (GAPDH) served as loading controls. −ADDT, no additive.

Supplemental Figure S4

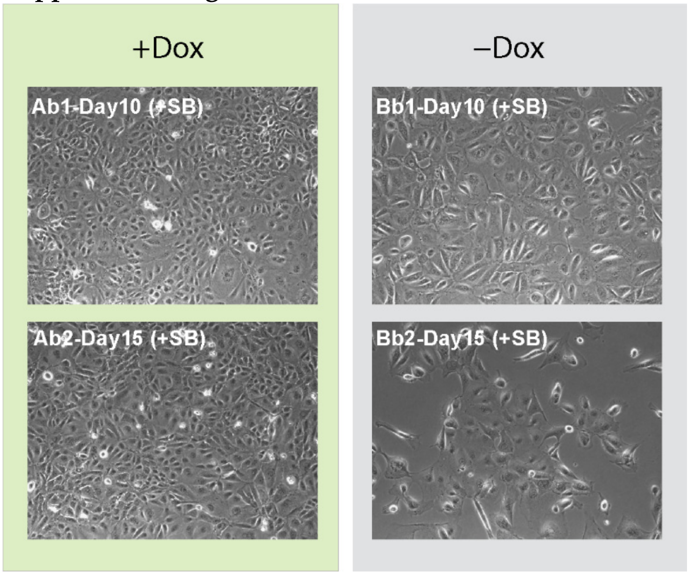

Supplemental Figure S4. Appearance of iEPDCs after long-term culture in the presence of SB with or without Dox. Representative phase contrast images of iEPDCs cultured in the presence of SB and with or without Dox as indicated in **Fig. 4A.** The images in the left panel display the morphology of actively proliferating iEPDCs in the presence of SB; the images in the right panel show the morphology of cell cycle-arrested iEPDCs in the presence of SB.

Supplemental Figure S5

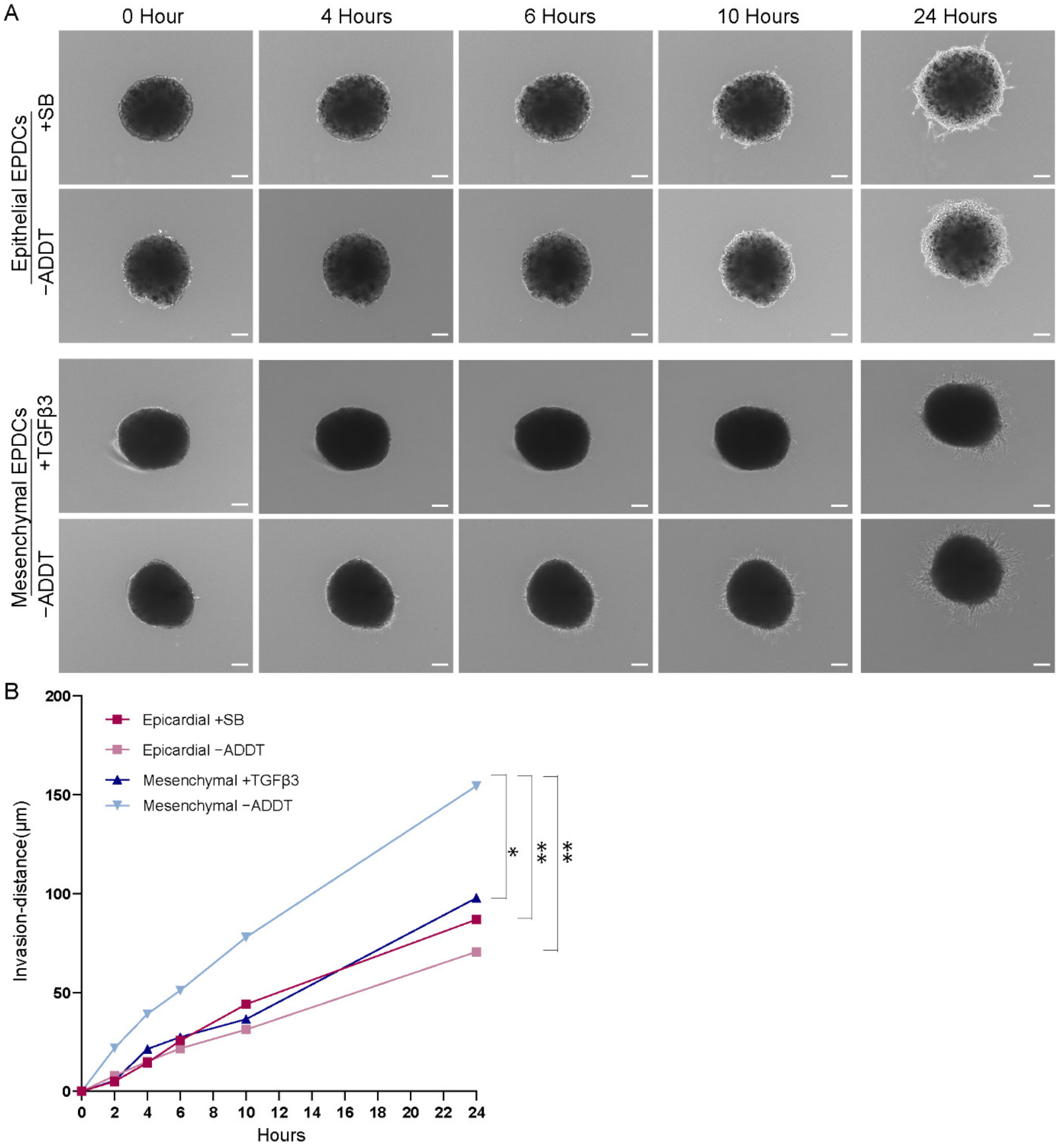

Supplemental Figure S5. Invasion ability of epithelial and mesenchymal primary human EPDCs. (**A**) Representative images of primary human EPDC (PD4) aggregates in a 3D collagen gel-based invasion assay. Scale bar = 100 μm. (**B**) Quantification of EPDC invasion distance within 24 hours. The assay was performed in Dox-free complete medium with the indicated additives using either epithelial iEPDCs (*i.e.* iEPDCs pretreated with SB [10 μM] to preserve their cuboidal epithelial morphology) or with mesenchymal iEPDCs (*i.e.* iEPDCs pretreated for 5 days with TGFβ3 [1 ng/ml] to induce a spindle-like morphology). *P<0.05, **P<0.01. −ADDT, no additive.

Supplemental Figure S6

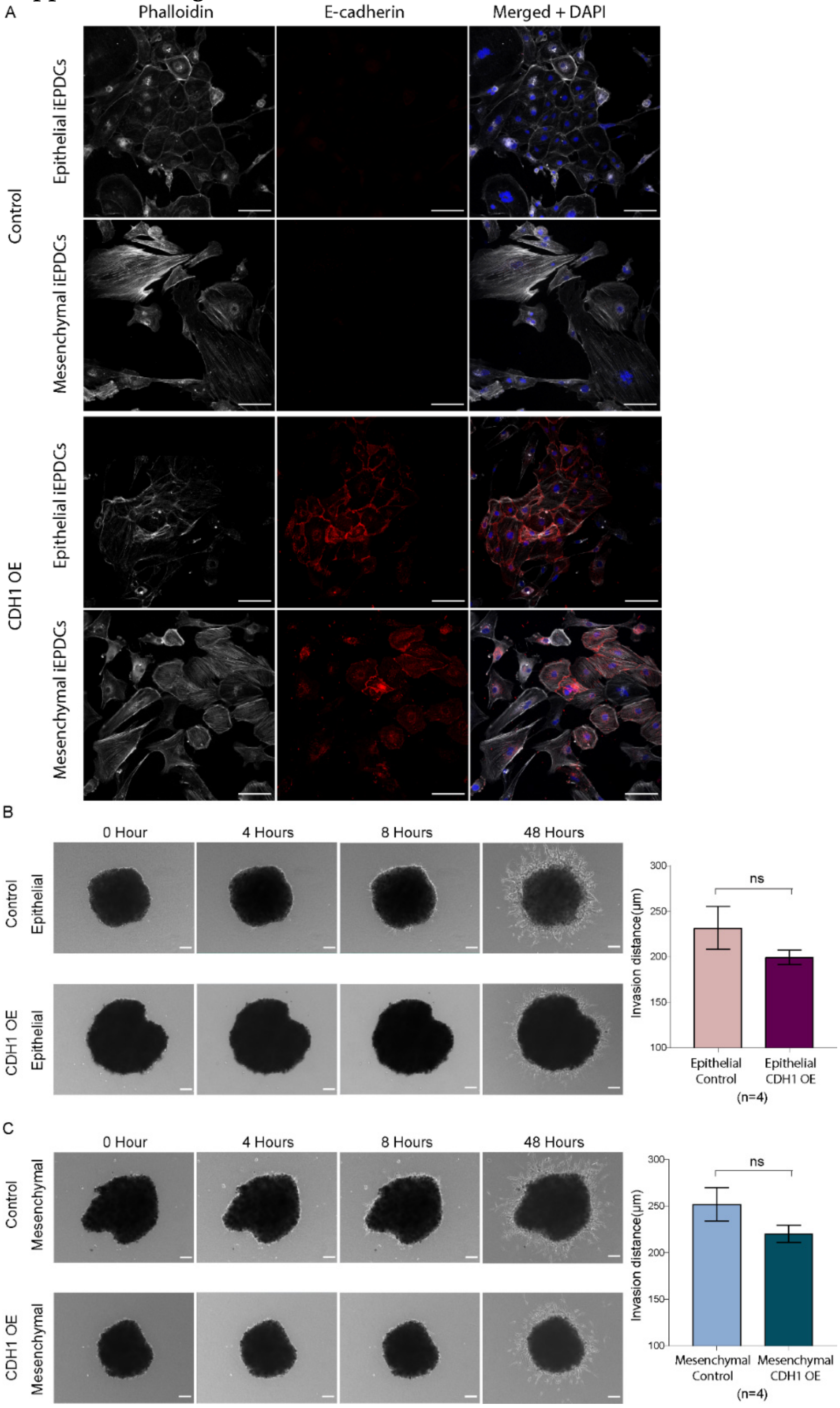

Supplemental Figure S6. CDH1 overexpression reduces the invasion ability of mesenchymal EPDCs. (**A**) Immunostaining showing robust E-cadherin expression in iEPDCs (PD41) following CDH1 overexpression (OE). Scale bar = 100 μm. (**B**) Left panel, representative images of epithelial iEPDCs overexpressing CDH1 in a collagen gel-based invasion assay. Right panel, quantification of epithelial iEPDC invasion distance within 48 hours. The epithelial iEPDCs were cultured in Dox-free complete medium with SB (10 μM), which was left out of the medium 24 hours before the start of the invasion imaging. Scale bar = 100 μm. (**C**) Left panel, representative images of mesenchymal iEPDCs overexpressing CDH1 in a collagen gel-based invasion assay. Right panel, quantification of mesenchymal iEPDC invasion distance after 48 hours. The mesenchymal iEPDCs were cultured in Dox-free complete medium with TGFβ3 (1 ng/ml) for 5 days before the start of the invasion imaging. Scale bar = 100 μm.
